# IgG4-related gastric disease with plasma cell-rich obliterative arteritis accompanied by early-stage gastric cancer: a case report

**DOI:** 10.1186/s40792-021-01126-6

**Published:** 2021-02-05

**Authors:** Masayoshi Obatake, Koichi Sato, Shigehiko Yagi, Hiromi Ohtani, Katsumi Kito

**Affiliations:** 1 Department of Surgery, Ehime Prefectural Central Hospital, 83 Kasugamachi, Matsuyama, Ehime 790-0024 Japan; 2 Department of Pathology, Ehime Prefectural Central Hospital, Ehime, Japan

**Keywords:** IgG4-related disease, Gastric cancer, Submucosal tumor, Obliterative arteritis

## Abstract

**Background:**

Immunoglobulin G4-related disease (IgG4-RD) is an immune-mediated inflammatory disorder that can involve multiple organs. It is characterized by IgG4-positive plasma cell-rich storiform fibrosis and obliterative phlebitis associated with a high serum IgG4 level. There are few reports of gastric IgG4-RD, especially those detected prior to systemic or other organ involvement.

**Case presentation:** A 70-year-old man was diagnosed with type 0–IIc gastric cancer at the anterior wall of the gastric corpus by upper gastrointestinal endoscopy. In addition, a submucosal tumor (SMT) 7 mm in diameter was found at the greater curvature of the angulus. Laparoscopic distal gastrectomy with regional lymph node dissection was performed. Pathology revealed a poorly differentiated adenocarcinoma in the type 0–IIc lesion and storiform fibrosis with infiltration of a large number of IgG4-positive plasma cells in the SMT. Postoperative laboratory testing showed elevation of serum IgG4 levels; thus, we diagnosed the SMT as IgG4-RD. Intriguingly, the gastric IgG4-RD lesion demonstrated IgG4-positive plasma cell-rich arteritis as well as typical obstructive phlebitis. The patient has been followed for 2 years after surgery without recurrence of cancer, but skin lesions of IgG4-RD have appeared.

**Conclusion:**

We report a rare case of IgG4-RD presenting as a gastric SMT, accompanied by early-stage gastric cancer. Our case may support a newly proposed relationship between IgG4-RD and malignancies. The gastric IgG4-RD lesion showed arteritis as well as obliterative phlebitis, potentially providing novel insight into IgG4-related vascular lesions.

## Background

IgG4-RD is an immune-mediated inflammatory disorder that involves multiple organs. It is characterized by IgG4-positive plasma cell-rich storiform fibrosis and obliterative phlebitis associated with a high serum IgG4 level [1]. Gastric IgG4-RD is a rare and newly recognized clinicopathologic entity that may occur as an isolated gastric lesion or as part of a multisystemic disorder [2]. We herein report a case of gastric IgG4-RD presenting as a submucosal tumor (SMT) and accompanied by early-stage gastric cancer. Intriguingly, the IgG4-related gastric lesion presented an unusual IgG4-positive plasma cell-rich obliterative arteritis without evidence of systemic vasculitis. This case may enlarge the morphologic spectrum of IgG4-related inflammatory vasculitis and support a relationship between IgG4-RD and malignancies.

## Case presentation

**Fig. 1:**
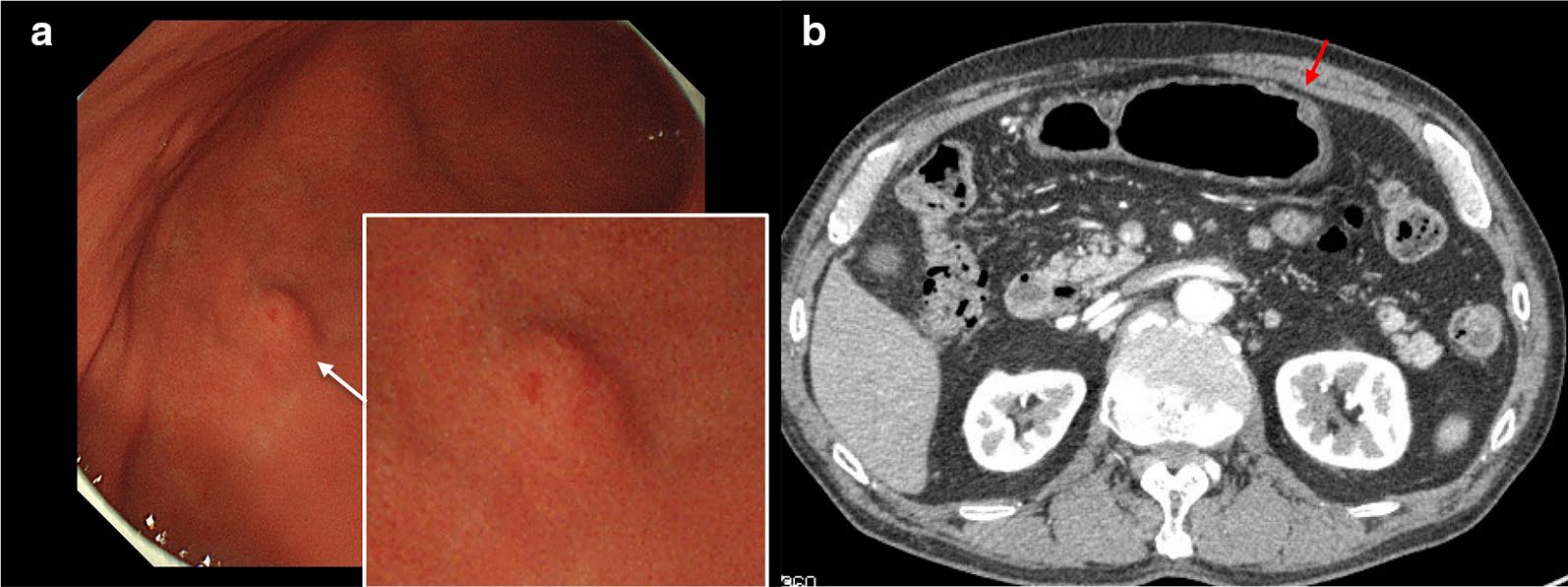
**a** An SMT 7 mm in diameter was revealed at the greater curvature of the angulus by upper gastrointestinal endoscopy. **b** The SMT was detected by contrast-enhanced CT

**Fig. 2:**
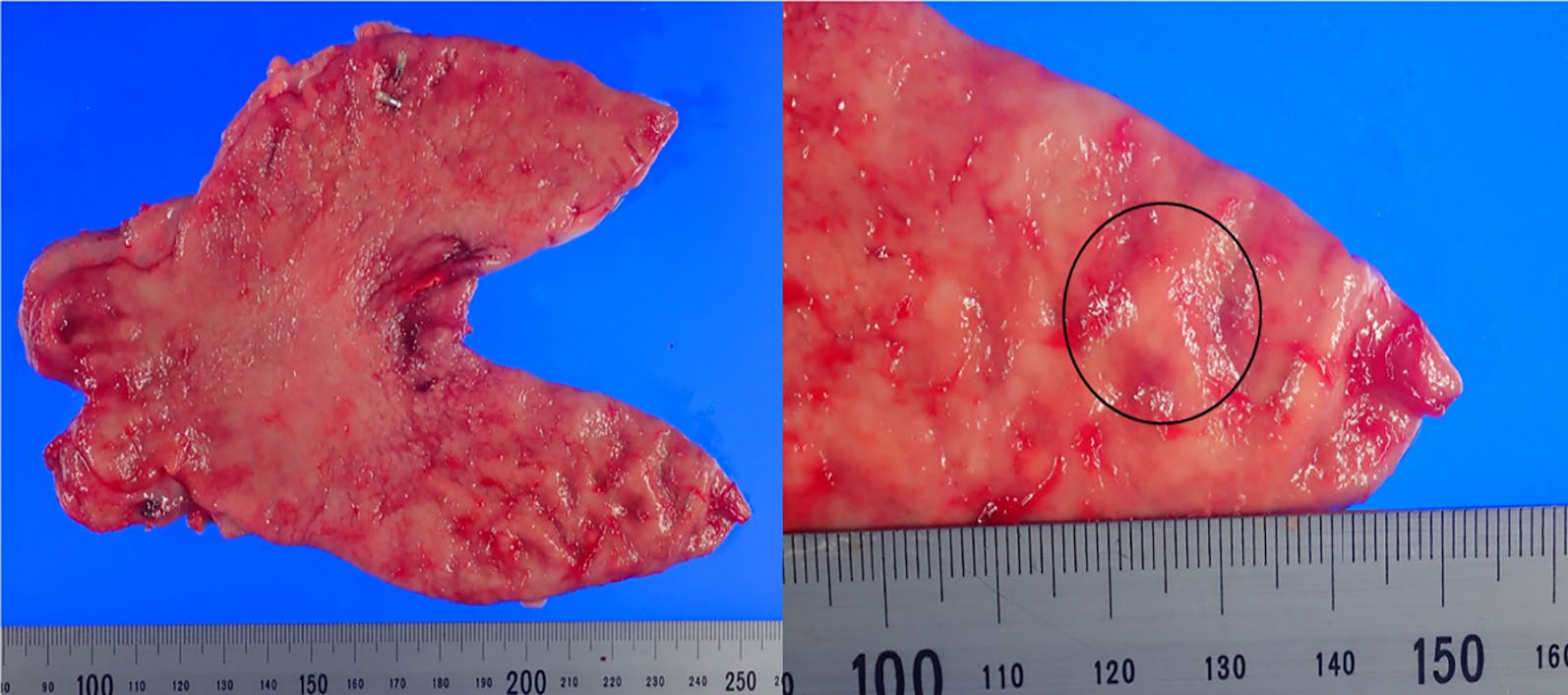
The resected specimen showed an SMT 7 × 5 mm in size

A 70-year-old man was diagnosed with type 0–IIc gastric cancer by upper gastrointestinal endoscopy and referred to our hospital for further examination. The type 0–IIc lesion was 30 × 15 mm in size and located at the anterior wall near the greater curvature of the gastric corpus. In addition to gastric cancer, a submucosal tumor (SMT) 7 mm in diameter was revealed at the greater curvature of the angulus (Fig. 1a), which was also detected by contrast-enhanced computed tomography(CT) (Fig. 1b). Laparoscopic distal gastrectomy with regional lymph node dissection was performed for both the gastric cancer and the SMT. The operation time was 299 min and the blood loss was 75 ml. The resected SMT lesion was 7 × 5 mm in size (Fig. 2).

**Fig. 3:**
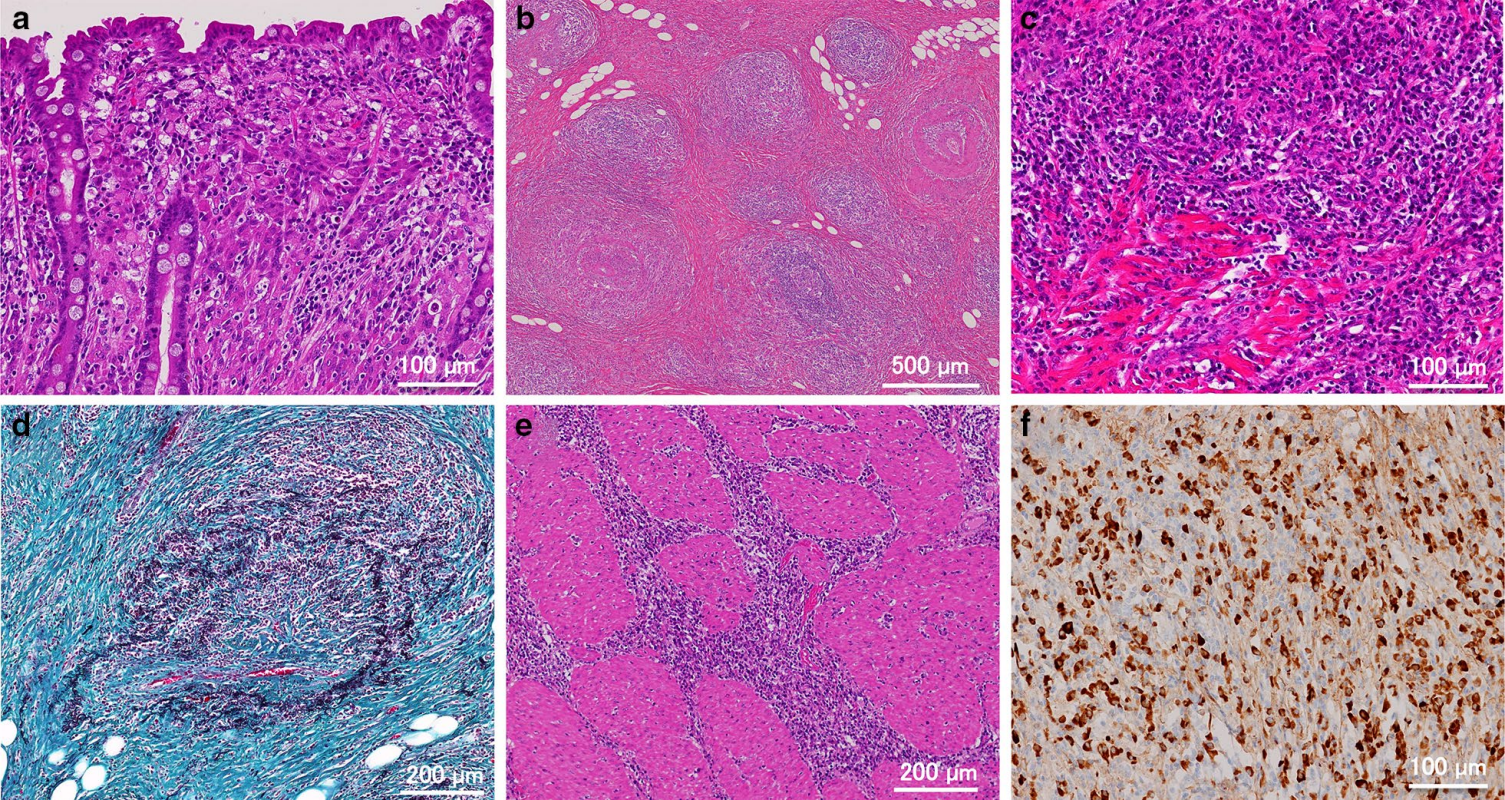
**a** Poorly differentiated adenocarcinoma with signet-ring cells had proliferated within the lamina propria (HE; original magnification, ×200). **b** The submucosal layer was expanded by fibrosis, lymphoid follicles, obliterative phlebitis, and arteritis (HE; original magnification, ×40). **c** A dense and diffuse infiltrate of plasma cells and lymphocytes (HE; original magnification, ×200). **d** Obliterative phlebitis is highlighted by Elastica staining (Elastica-Masson; original magnification, ×100). **e** Lymphoplasmacytic infiltrate is observed in the interstitium of the muscularis propria (HE; original magnification, ×100). **f** Immunostaining for IgG4 reveals infiltration by a large number of IgG4-positive plasma cells (original magnification, ×200)

Pathology revealed the type 0–IIc lesion to be poorly differentiated adenocarcinoma with signet-ring cells (Fig. 3a). The cancer cells were localized in the mucosal layer, and no lymph node metastasis was identified. The SMT at the posterior wall was covered by normal gastric mucosa, but the submucosal layer was expanded by a dense and diffuse infiltrate of plasma cells and lymphocytes associated with lymphoid follicles, storiform fibrosis and obliterative phlebitis (Fig. 3b–d). The interstitium of the muscularis propria and the bottom of the mucosa also showed plasma cell and lymphocyte infiltrates (Fig. 3e). Immunostaining for IgG and IgG4 showed abundant IgG-positive plasma cells, of which over 80% were IgG4-positive. The number of IgG4-positive cells exceeded 200 per high-power field (Fig. 3f). These findings were consistent with the histological criteria for IgG4-RD. In addition to plasma cells, an elevated number of eosinophils, immunoglobulin E (IgE)-positive mast cells, and forkhead box P3 (FOXP3)-positive regulatory T-lymphocytes showed infiltration (data not shown).

Further histological examination of the resected tissue revealed several IgG4-related inflammatory foci discontinuously distributed throughout the stomach. They were predominantly located in the submucosa and muscularis propria, occasionally causing wall thickening. Regional lymph nodes also contained fibrotic foci with lymphoplasmacytic infiltrate, occasionally with obliterative phlebitis.

**Fig. 4:**
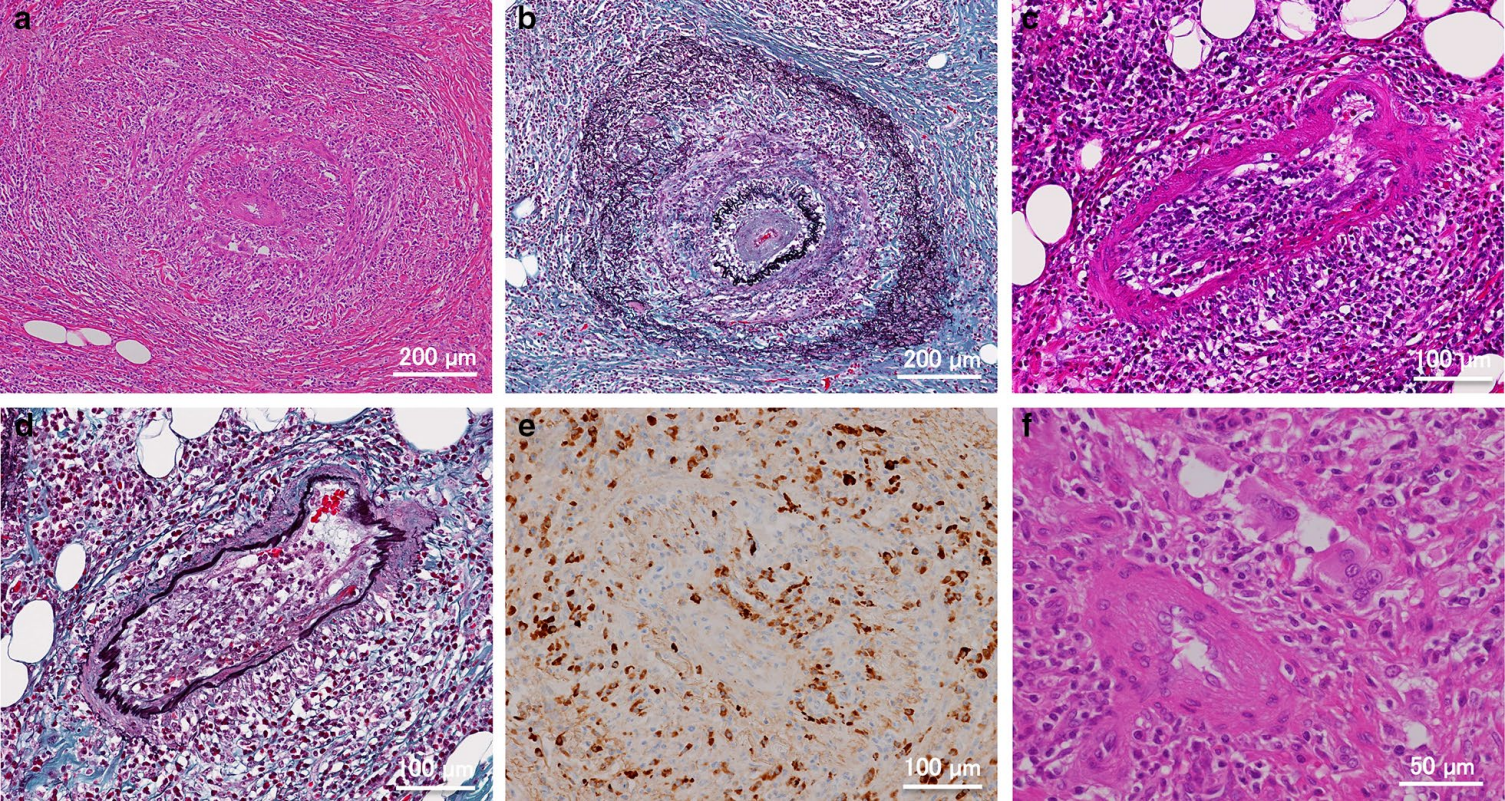
**a**, **c** Obliterative arteritis of a small-sized artery shows abundant lymphoplasmacytic infiltrate within the expanded intima and media (HE; original magnification, **a** ×100, **c** ×200). **b**, **d** Elastica staining is highlighted in the elastic membranes of the artery (Elastica-Masson; original magnification, **b** ×100, **d** ×200). e Immunostaining for IgG4 shows IgG4-positive plasma cells infiltrate in the arterial wall (original magnification, ×200). **f** Multinucleated giant cells were observed in the inflamed arteries (HE; original magnification, ×400)

In addition to obliterative phlebitis, small-sized arteries were thickened and narrowed with marked intimal and medial inflammation by lymphoplasmacytic infiltrate (Fig. 4a–d). The arterial endothelium and smooth muscle appeared to be damaged, but without evidence of neutrophil infiltration, fibrinoid necrosis, or thrombus. Numerous IgG4-positive plasma cells were identified within the intima and media of inflamed arteries (Fig. 4e). Multinucleated giant cells were also observed (Fig. 4f).

A postoperative laboratory test revealed elevation of the serum IgG4 level (1,178 mg/dl), with no increase in myeloperoxidase anti-neutrophil cytoplasmic autoantibodies (MPO-ANCA), proteinase 3(PR3)-ANCA, or interleukin 6 (IL-6). There was no clinical evidence of systemic vasculitis.

The postoperative course was uneventful, and the patients were discharged on the 15th postoperative day. The patient has been followed for 2 years after surgery without recurrence of cancer, during which time only skin lesions of IgG4-RD have appeared. Cutaneous IgG4-related lesions have shown repeated exacerbations and remissions despite corticosteroid treatment.

## Discussion

Multiple lines of evidence indicate that IgG4-RD is an autoimmune condition; however, the exact pathogenesis remains incompletely understood. There is an emerging consensus that the IgG4 antibodies in IgG4-RD are not pathogenic, and that T cells, especially CD4+ cytotoxic and T-follicular helper cells, have a more important role [3]. Elevations in serum and tissue IgG4 concentrations are not specific to IgG4-RD; they are also found in several other disorders such as multicentric Castleman disease, eosinophilic granulomatosis with polyangiitis, sarcoidosis and allergic diseases [4]. Thus, histopathology is the key to the diagnosis of IgG4-RD. According to the consensus statement, there are four criteria by which IgG4-RD can be recognized in previously unrecognized organs: (1) characteristic histologic findings (notably storiform fibrosis and obliterative phlebitis), numerous IgG4-positive cells and an elevated IgG4/IgG-positive cell ratio; (2) an elevated serum IgG4 level; (3) response to corticosteroids; (4) coexistence of other organs with IgG4-RD involvement [5].

In our case, gastric IgG4-RD was discovered during the preoperative examination for gastric cancer. Recent data have suggested that a history of IgG4-RD may be a risk factor for malignancies. In their analysis of 106 cases of IgG4-RD, Yamamoto et al. reported that IgG4-RD patients had a 3.8-fold elevated risk of malignancies compared to same-age cohorts [6]. Another retrospective study showed that history of malignancy was 2.5 times more likely in IgG4-RD patients compared to the general public [7]. Notohara et al. reported that two out of six cases highly suggestive of IgG4-related gastrointestinal disease were accompanied with intramucosal gastric adenocarcinoma [2]. Finally, a very recent prospective cohort study demonstrated that IgG4-RD patients have a 2.78-fold increased risk of malignancy over a median of 61.4 months of follow-up, with the most common malignancy site being the gastrointestinal tract [8]. These findings suggest that IgG4-RD may be a paraneoplastic syndrome in some patients. However, no studies have directly compared the incidence of gastric cancer between patients with IgG4-RD and risk-factor-matched controls; this remains important work for future prospective studies.

Although the mechanism underlying the association of IgG4 and carcinogenesis is not fully understood, several findings have been reported on how IgG4 is involved in cancer development. IgG4 was found to positively correlate with regulatory T cells and to negatively correlate with cytotoxic T cells, supporting its involvement of immune tolerance in cancer [9]. Karagiannis et al. revealed that tumor-specific IgG4 was produced locally in the tumor microenvironment and that IL-4 and IL-10 expression was enhanced [10]. Wang et al. demonstrated that increased IgG4 in cancer microenvironment bound to cancer-specific IgG1 and inhibited the subsequent immune effector response that would detect and destroy cancer cells [11]. In fact, the elevated levels of tissue and serum IgG4 have been observed in several kinds of malignancies, including gastric cancer, potentially associated with an unfavorable prognosis [12, 13].

Vascular involvement of IgG4-RD frequently occurs in the aorta and branching medium-sized arteries, such as the coronary arteries, superior mesenteric artery, and iliac arteries; by contrast, small-sized peripheral arteritis is rare [14]. IgG4-related vasculitis is characterized by adventitia-dominant inflammation, whereas regular vasculitis generally affects the intima and media. There are only a couple of reports addressing IgG4-related arteritis in small-sized arteries [15, 16]. To our knowledge, ours is the first case report of IgG4-related obliterative arteritis in the stomach.

Other forms of vasculitis, such as polyarteritis nodosa and ANCA-associated vasculitis can be considered differential diagnoses of this disorder. They are necrotizing vasculitis of medium or small arteries with neutrophil infiltrates and fibrinoid necrosis. Our case lacked the clinical features of systemic vasculitis with no increase in ANCA; the inflamed arteries were almost exclusively localized in the SMT where inflammation was most prominent. There is the possibility that obliterative arteritis may be a secondary arterial involvement of interstitial inflammation.

It has been reported that gastric IgG4-RD lesions are mainly localized in the muscularis propria and are often difficult to diagnose by endoscopic biopsy [2]. When the submucosal lesions increase in size or become symptomatic, endoscopic ultrasound-fine needle aspiration (EUS-FNA) should be adopted to make a histological diagnosis, or various endoscopic resection procedures should be considered to allow treatment. The gastric IgG4-RD does not always appear as SMT, but is also known to form ulcers or wall thickening [2]. When other organ involvement and systemic symptoms are not clinically evident, a definitive diagnosis of gastric IgG4-RD by endoscopy might be difficult, but IgG4-RD should always be kept in mind for differential diagnosis of gastric lesions.

## Conclusion

We report a case of a 70-year-old man with IgG4-related gastric disease presenting as a submucosal tumor and early-stage gastric cancer. Our case may support the newly proposed relationship between IgG4-RD and malignancies. The IgG4-related gastric lesion showed arteritis as well as obliterative phlebitis, potentially providing novel insight into IgG4-related vascular lesions.

## Data Availability

Data sharing is not applicable to this article as no datasets were generated or analyzed during this case report.

## Abbreviations

IgG4-RD: Immunoglobulin G4-related disease
SMT: Submucosal tumor
CT: Computed tomography
IgE: Immunoglobulin E
FOXP3: Forkhead box P3
MPO-ANCA: Myeloperoxidase anti-neutrophil cytoplasmic autoantibodies
PR3: Proteinase 3
IL-6: Interleukin 6
EUS-FNA: Endoscopic ultrasound-fine needle aspiration
